# Life experience rather than domestication accounts for dogs’ increased oxytocin release during social contact with humans

**DOI:** 10.1038/s41598-021-93922-1

**Published:** 2021-07-13

**Authors:** Gwendolyn Wirobski, Friederike Range, Franka S. Schaebs, Rupert Palme, Tobias Deschner, Sarah Marshall-Pescini

**Affiliations:** 1 Domestication Lab, Wolf Science Center, Konrad Lorenz Institute of Ethology, Department of Interdisciplinary Life Sciences, University of Veterinary Medicine, Veterinaerplatz 1, 1210 Vienna, Austria; 2 Interim Group Primatology, Max Planck Institute for Evolutionary Anthropology, Deutscher Platz 6, 04103 Leipzig, Germany; 3 Unit of Physiology, Pathophysiology and Experimental Endocrinology, Department of Biomedical Sciences, University of Veterinary Medicine, Veterinaerplatz 1, 1210 Vienna, Austria; 4 University of Leipzig, ZLS, Prager Str. 34, 04317 Leipzig, Germany

**Keywords:** Coevolution, Social evolution, Psychology, Animal behaviour, Animal physiology

## Abstract

Dogs’ increased human-directed sociability compared to wolves may be the result of increased oxytocin system activity and decreased stress responses, but comparative studies accounting for life experience are lacking. We compared hand-raised, pack-living wolves’ and dogs’ behavior and hormone concentrations after interacting with a closely bonded and a familiar human. Both preferred the bonded partner, but dogs showed less variability in human-directed sociability than wolves. Physical contact was not associated with oxytocin but correlated positively with glucocorticoids in the pack-living animals when the human was not bonded. To clarify the role of life experience, we tested pet dogs and found that oxytocin concentrations correlated positively with physical contact with their owners, while glucocorticoids remained unaffected. Results show that, given similar experiences, wolf-dog differences in human-directed sociability and associated hormones are subtle and indicate that factors related to life as a pet dog rather than domestication account for oxytocin release during human–dog interactions.

## Introduction

Domesticated compared to wild-type animals are thought to show reduced fear and increased willingness to interact with humans (Emotional Reactivity and Selection for Tameness hypotheses:^1^,^2^; Hypersociability hypothesis:^3^; Table 1). Such changes are thought to be associated with an altered hypothalamo-pituitary-adrenal (HPA) axis and oxytocinergic system (re)activity resulting in a diminished stress response and greater inclination to approach humans (‘Domestication Syndrome’:^4,5–9^).

**Table 1:** Dog domestication hypotheses presented and tested in the current study.

Domestication hypothesis	Synopsis	References
Emotional reactivity (Selection for Tameness) hypothesis	Dogs' enhanced human-directed social skills compared to wolves, evolved as a by-product of selection for decreased (human-directed) fear- and aggression and increased docility	Hare and Tomasello^1^; Trut et al.^2^
Hypersociability hypothesis	Structural variants in two genes of the dog genome associated with Williams-Beuren syndrome in humans, which are absent in wolves, contribute to dogs' exaggerated motivation to seek social contact with humans	vonHoldt et al.^3^
Two Stage hypothesis	Rather than resulting from selection during domestication alone, experiences with humans during ontogeny affect dogs' behavioral and physiological responses to social contact with humans later in life	Udell et al.^34^; Udell and Wynne^35^

Pet dogs^10,11^; reviewed in^12^, but also extensively human-socialized wolves are capable of forming long-lasting affiliative relationships^13–16^ with their caregivers. However, in support of dogs being more sociable towards humans than wolves, in previous studies, pet dogs approached a familiar person faster and spent more time in close proximity to them than human-socialized wolves^3,17^. Similarly, socialized pack-living dogs spent longer in proximity and looking at the human in a training session^18^ and in body contact with a bonded experimenter during a ‘cuddle session’ than wolves^19^. In terms of the suggested hormonal correlates, increased oxytocin and/or decreased glucocorticoid secretion is frequently reported following affiliative dog-owner interactions in both canine and human participants^20–26^; but see^27–29^, but effects may be mediated by interaction style, type of touch, as well as the quality of the relationship with the interaction partner^24,30–32^.

Studies directly comparing changes in hormonal concentrations of wolves and dogs when interacting with humans are sparse, with two notable exceptions. In the first study on similarly raised and kept dogs and wolves, both showed a decrease in salivary glucocorticoid concentrations following a short training session with their caregiver^18^. The second study^7^ found that pet dogs’ increased urinary oxytocin concentrations were linked to mutual eye gazing with their owners, but wolves’ were not, and that owners’ urinary oxytocin change ratios correlated with their dogs’. However, this effect was only present in a subset of pet dogs that gazed particularly long at their owners, and not in short-gaze dogs. Based on the wolf-dog comparison, the authors proposed a co-evolved feedback loop between dogs’ and humans’ oxytocinergic systems, providing an interesting framework for the role of said system during dog domestication. However, it has been argued that comparing pet dogs to enclosure-living wolves could have biased the results of the latter study^33^. Indeed, the importance of controlling for previous experiences and exposure to humans in sociability testing cannot be emphasized enough (Two Stage hypothesis:^34,35^, Table 1;^15,19,36,37^. In addition, it is likely that the bond between a pet dog and its owner differs qualitatively from the relationship an enclosure-living animal may establish with its caregiver^38^. This is supported by data showing that oxytocin and glucocorticoid concentrations are strongly mediated by previous experience with the interaction partner (familiarity) and quality of the relationship^32,39^.

To help clarify the hormonal mechanisms of human-directed social contact seeking in wolves and dogs, we conducted two consecutive experiments:

### Experiment 1: Pack-living dogs and wolves

In Experiment 1, we compared contact seeking behavior (sociability), self-directed behaviors (SDB) e.g. yawning, lip licking, head and body shaking (behaviors which have been associated with fear and stress in dogs ^40,41^, and urinary oxytocin and glucocorticoid metabolite (OTM and GCM, respectively) concentrations of comparably hand-raised, pack-living dogs and wolves—i.e., having had the same general experiences with humans across their lifetimes—at the Wolf Science Center (WSC), Ernstbrunn, Austria. Dogs and wolves were tested with two human partners of different relationship strengths (i.e., their hand raisers and a familiar person) in a dyadic social interaction test and two control conditions (food, resting/’baseline’; Table 2). Considering a previous study^7^ found an increase in dog but not wolf owners’ oxytocin concentrations following interactions we also analyzed the oxytocin concentrations of the human partners before and after interacting with the dogs and wolves. Based on the Hypersociability hypothesis^3^, previous results linking dog domestication and social touch to increased oxytocinergic system activity^7,42–47^, and the finding that oxytocin concentrations are mediated by relationship strength^32^, we predicted longer contact times and higher OTM concentrations in dogs than wolves and when interacting with the bonded compared to the familiar partner. The Emotional Reactivity hypothesis(^1,2^; Table 1) predicts that dogs would approach and interact more with the human partners (regardless of relationship strength) than wolves. In addition, wolves should show more SDBs and higher GCM concentrations, than dogs during close contact with humans. Yet, previous studies with equally socialized wolves and dogs showed a decrease in salivary glucocorticoid concentrations following a training session with a bonded caretaker in both species^18^. Therefore, we predicted lower urinary GCM concentrations following the social interaction in both species, mediated by the relationship with the human partner. We further predicted higher OTM concentrations in the human participants following the interaction with a wolf or dog than the control conditions.

**Table 2:** Experimental test and control conditions.

Condition	Focal subjects	Short description
Baseline	Wolves, pack dogs, pet dogs	Animals resting in home enclosure with pack mates present (pet dogs: resting in familiar indoor environment)
Dyadic social interaction (familiar human partner)	Wolves, pack dogs, pet dogs, humans	Familiar human initiates an interaction with the animal at the enclosure fence
Dyadic social interaction (bonded human partner)	Wolves, pack dogs, pet dogs, humans	Bonded human (i.e., the hand raiser for pack dogs and owner for pet dogs) initiates an interaction with the animal at the enclosure fence
Food control	Wolves, pack dogs, pet dogs	Food provided by a familiar person (i.e., an animal keeper not involved in hand raising or the other conditions), without physical contact
Animal present, no interaction	Humans	Control condition at the enclosure, no interaction with the animal (person facing away from animal)
Animal not present	Humans	Control condition, human participant performing office work

### Experiment 2: Pet dogs

In Experiment 2, we tested a group of pet dogs using the same paradigm (i.e., dyadic social interaction tests with their owner and a familiar person, food control, baseline; Table 2) to clarify the role of life experience for behavioral and hormonal responses to human social contact. The Two Stage hypothesis(^34,35^; Table 1), posits that an animal’s human-directed behavior depends crucially on previous experiences during ontogeny. Hence, we predicted that the pet dogs would spend more time with and show higher OTM concentrations after interacting with the bonded partner (i.e., their owner) than the familiar person. Finally, in the pet dogs, we predicted lower GCM concentrations after the social than the control conditions, also mediated by relationship strength.

## Materials and methods

### Experiment 1: Pack-living dogs and wolves

#### Study site and animals

We tested 10 grey wolves and 11 mongrel dogs (Table S1) housed at the Wolf Science Center (WSC, www.wolfscience.at/en/), Ernstbrunn, Austria, in a within subject design. All animals were hand-raised by animal professionals and separated from their mothers in their first week of life, experiencing continuous (24 h) contact with humans in the first 4–5 months of their life. After this period, regular positive reinforcement training sessions and behavioral or cognitive testing ensured that all animals experienced comparable levels of positive contact with humans throughout their lives. All dogs and wolves were housed in dyads or groups of up to four animals in large outdoor enclosures. In accordance with their species-specific requirements, dogs were fed daily with commercial dog kibble and wolves were provisioned with raw meat and carcasses of deer, rabbit, and chicken, 3–4 times a week. Data collection took place from May to early December 2017 and late April to November 2018, avoiding the wolf breeding season and its associated behavioral and hormonal changes. All male dogs and wolves were vasectomized aged approx. 6 months to prevent reproduction while maintaining their full endocrine and behavioral profiles. Female dogs were not sampled if they were in heat and male dogs were not sampled if their pack included a female in heat. In such cases, we waited until all behavioral and external physiological signs of the heat had disappeared before resuming testing.

#### Human participants

Twelve adult, female participants who either worked at the WSC as animal trainers (i.e., who were hand raisers of the animals, thus acting as the bonded interaction partners; N = 5) or as researchers (i.e. familiar people who did not have direct contact with the animals and were never involved in hand raising, thus acting as the familiar interaction partners for the animals; N = 7) volunteered to participate in the study. The perception of a stronger bond with the animal following the hand-raising experience is confirmed by the fact that animal trainers working at the WSC consistently rated their relationships with a particular animal higher if they were directly involved in its hand raising than if they were not^48^.

#### Experimental design

To compare behavioral and hormonal responses of pack-living dogs and wolves following close social contact with different human partners, all animals were tested in four conditions: (1) a ‘baseline’ (i.e., the focal animals were resting in their home enclosures), (2) a dyadic social interaction test with a familiar human partner, (3) and with a bonded human partner, and (4) a food control (Table 2). Comparing the social interaction conditions with the food control allowed us to evaluate whether physical interaction per se was responsible for specific hormonal changes. Comparing the two social conditions allowed us to assess if oxytocin and glucocorticoid release were mediated by physical contact time and/or relationship strength with the human partner.

**Figure 1:**
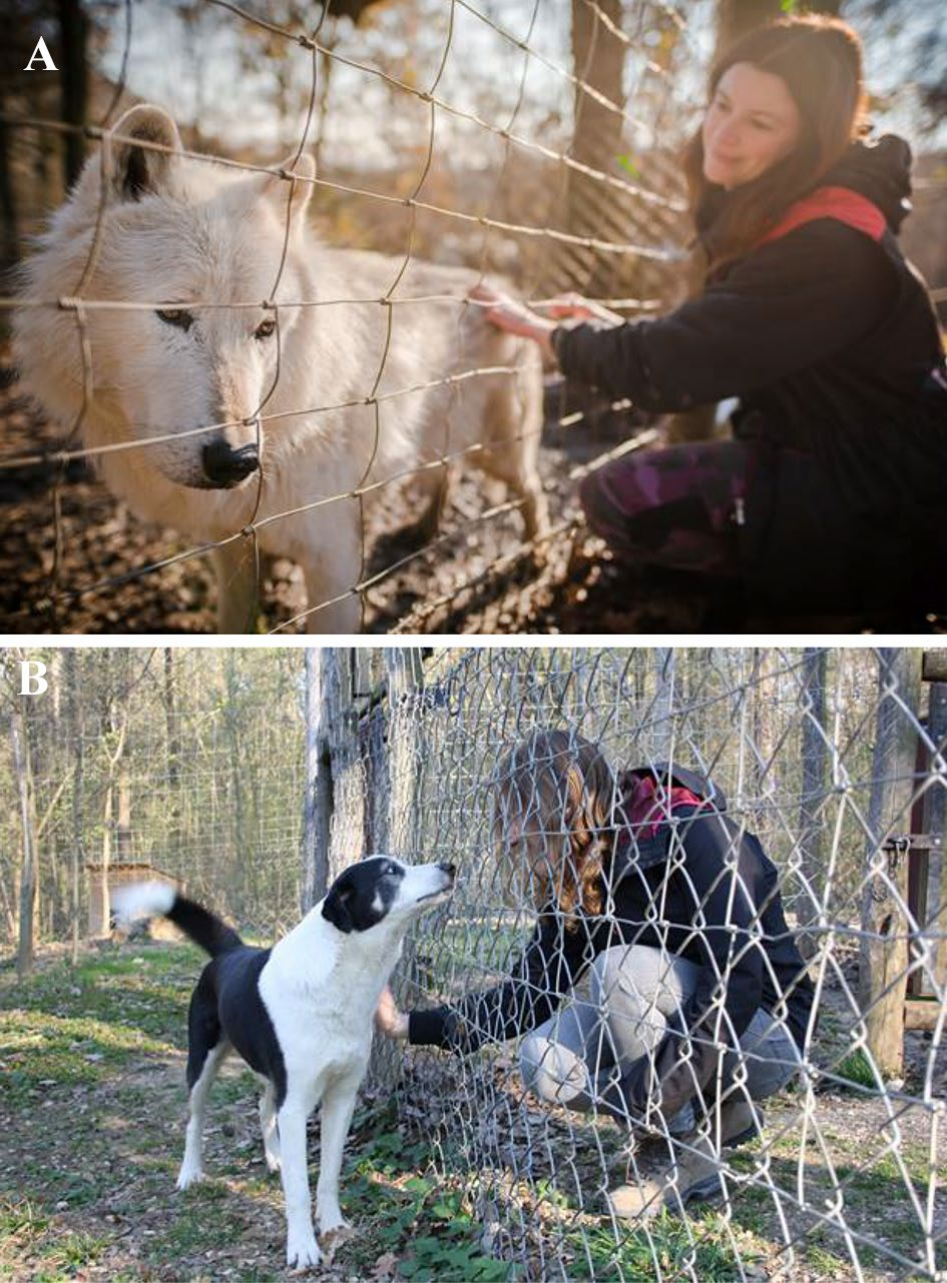
(**A**,**B**) Dyadic social interaction test in the animal’s home enclosure. Interaction test with a human partner with (**A**) a wolf, (**B**) a pack-living dog.

Testing was conducted in a semi-randomized and counterbalanced order. The interaction tests involved a ‘cuddle session’ which lasted 5 min and during which animals stayed in their familiar home enclosures and were free to approach the fence to be petted by the human partner (Fig. 1 A-B; Movies S1 and S2). This was followed by a short training session, during which animals were asked to respond to known commands in exchange for a food reward (15 pieces of sausage, 1 × 1 × 1 cm). The exact same amount of food was given to the animals in the food control by a third person (an animal keeper who was not involved in hand raising and did not participate in the dyadic social interaction tests), but without any physical interaction or verbal appraisal. Prior to testing, focal animals were not fed and did not participate in other tests or activities for at least 2 h. Each test was preceded by 60 min of behavioral observations of the focal animals to ensure no major disruptions occurred which could have affected subsequent hormonal measures. If any disruptions (e.g., aggression within the pack, playing and grooming bouts, or external stimuli such as passing visitors, trainers, students, or researchers, that caused an observable behavioral change in the focal pack) occurred, the testing was re-scheduled for another day. Each animal was tested only once per day and provided two baseline samples on separate occasions.

Human participants were tested with at least one wolf or one dog and in the respective control conditions (animal present, no interaction; animal not present) (Table 2). The ‘animal present, no interaction’ control was analogous to the interaction condition but no interaction (no touching, talking or gazing) between the human and the animal took place. The human participant walked to the animal’s home enclosure together with the experimenter where she sat down quietly in the air lock compartment, facing away from the animal to avoid eye contact. She stayed there for the same duration as the interaction lasted in the social interaction condition. Following a 60 min waiting period during which she was not allowed to interact with other humans or animals (computer work, reading, or resting was allowed), she donated the post urine sample. For the ‘animal not present’ control, the human participant provided a pre-test urine sample, and then proceeded to work on a computer, read, or rest for 60 min, without any contact to animals or humans. Then she donated the post urine sample.

#### *Behavioral data collection*

Social interaction tests were filmed, and videos were subsequently coded with Solomon behavioral coding software (version beta 17.03.22, copyright András Péter, https://solomon.andraspeter.com) using a canid ethogram (Table S2). To control for small variation in the total duration of the interaction session and varying amounts the animals spent visible on the videos (i.e., all subjects were free to move around in the enclosures during testing while the camera was positioned on a fixed spot), we first calculated the ‘total time in sight’ by subtracting the time spent out of sight from the total duration of the session and then normalized durations of the time spent in body contact (duration in body contact/total time in sight) and the rate of self-directed behaviors (SDB) per second (sum of yawning, lip licking, and head or body shakes/total time in sight) for the ‘total time in sight’ for further statistical analyses. We subsequently refer to the ‘total time in sight’ as the ‘interaction time’. Inter-observer reliability (IOR) testing was conducted on 20% of the interaction videos scored by two independent observers using the package irr (version 0.84.1) in R, version 4.0.2^54^. This revealed very high reliability for all behaviors: Duration spent in proximity to human partner, interclass correlation coefficient (ICC) = 0.99, P < 0.01, 95% confidence intervals (CI) 0.92–0.99; duration spent in body contact with human partner, ICC = 0.99, P < 0.01, CI 0.79–0.99; sum of self-directed behaviors, ICC = 0.93, P < 0.01, CI 0.47–0.99.).

#### Urine sample collection and hormone measurement

Spontaneously voided urine samples were collected non-invasively from all dogs and wolves during leashed walks using an expandable metal stick with a plastic cup attached to it. Prior to testing all animals were habituated to the urine sampling procedure. All animals were taken on urine collection walks 60 min after testing and we collected the first urine they voided. Samples used for analysis were collected on average 74 min after testing (SD 8.5 min, range 60–104 min; comparable to previous studies:^29,44,50^). The baseline samples were collected following at least 60 min of undisturbed resting in the familiar home enclosures. Human urine samples were taken by the participants themselves immediately before and 60 min after the interaction test.

Upon collection, all samples were split into four 1 ml aliquots. 100 µl of a 0.1% phosphoric acid (PA) was added to two aliquots to prevent oxytocin degradation in the samples and optimize conditions for storage^49–52^. One 1 ml aliquot of each sample was kept without PA to measure urinary specific gravity (SG; to account for urine dilution in wolf and dog samples), urinary creatinine (crea; to control for urine dilution in human samples) and glucocorticoids. All samples were frozen at −20 °C within 15 min of collection. Solid-phase extractions (SPE) following a previously validated protocol was performed for urinary OTM measurement^51^, and diethyl-ether extractions for urinary GCM^50^. Extracted samples were sealed and shipped on dry ice to the Max-Planck-Institute for Evolutionary Anthropology, Leipzig, Germany, for OTM measurement, and to the Unit of Physiology, Pathophysiology and Experimental Endocrinology, University of Veterinary Medicine, Vienna, Austria, for GCM measurement. For urinary OTM measurement, we used a commercially available enzyme-immunoassay (EIA) kit (Arbor Assays, Ann Arbor, Cat.No: K048-H5). The assay was analytically and physiologically validated for our study species and sample matrix^52^. The assay standard curve ranged from 16.38 to 10 000 pg/ml and assay sensitivity was 17.0 pg/ml. Intra-assay coefficients of variation (CVs) were 8.6% (dog/wolf samples) and 9.5% (human samples). Inter-assay CVs of high and low concentration quality controls (QCs) were 9.7% (high) and 12.4% (low). For urinary GCM measurement, we used an in-house cortisol EIA with an antibody produced against cortisol-21-HS:BSA, previously validated for our purpose^50^. The assay standard curve ranged from 2 to 200 pg/well and assay sensitivity was 2 pg/well. Intra- and inter-assay CVs were 5.3% and 7.5%, respectively. All samples were measured in duplicates and repeated if optical density (OD) values differed more than 10%. Wolf and dog urinary OTM and GCM concentrations were corrected for variable water content of the samples using urinary SG, measured with a digital refractometer, as previously described^50–52^. SG corrected hormone concentrations are expressed as OTM pg/ml SG and GCM ng/ml SG^53^. Human urinary OTM concentrations were expressed as pg/mg crea to account for variable concentration and volume of the voided samples.

### Experiment 2: Pet dogs

#### Experimental design

The surprisingly few differences in behavioral and hormonal responses to social interactions with humans of pack-living dogs and wolves^18^ strikingly contrast with recent findings^7^ suggesting the latter may have been biased by the use of pet dogs instead of animals with similar life experiences. Hence, we conducted a second experiment with pet dogs using the same test paradigm: Ten female, adult dog owners and their pet dogs (5 males and 5 females; mean (SD) age 6.9 (4.1) years; Table S8) were recruited from the environment of the researchers and volunteered to participate in the current study. Pet dog-owner dyads were chosen on the basis that they were highly familiar with the testing environment at the WSC to keep conditions as similar and comparable as possible to the pack-living animals. Female colleagues who were familiar with the dogs (i.e., had interacted with them before on multiple occasions) were recruited to act as familiar interaction partners for the pet dogs. All dogs had lived with their owners for at least 6 months prior to the start of the study and continued to do so after the study ended.

To keep procedures consistent with Experiment 1, we tested the pet dogs outdoors in two settings: (1) in an enclosure-type setting with a fence between the human partner and the dog, and (2) in an open space, without a fence, because we hypothesized that the presence of a fence may affect the dogs’ interest in approaching and staying close to the humans, particularly in pet dogs who are not used to this mode of interaction. The test locations were familiar to the pet dogs and they were given several minutes to explore their surroundings before testing started. All pet dogs were tested in both settings (i.e., with/without the fence), with their owners, a familiar person, and in the food condition. One dog completed only four tests due to owner time constraints and unavailability of the dog for further tests. The baseline measure was taken following a 60 min resting period in their familiar environment (i.e., the owner’s office). The social interaction tests and food control were performed analogous to Experiment 1. To eliminate possible order effects, testing was conducted in a semi-randomized and counterbalanced order across subjects, and each pet dog was tested only once per day. The dogs were not fed, walked, or otherwise interacted with during the 2 h prior to the start of the test. Each test was preceded by a 60 min resting period in their familiar environment with the owner present but instructed not to interact with the dog.

Behavioral and hormonal data collection and sample treatment was identical to Experiment 1, as described above.

### Statistical analyses

We fitted generalized and linear mixed models (GLMM, LMM) in R statistical software^54^, version 4.0.2, https://www.R-project.org), using the function ‘lmer’ of the package lme4 (version 1.1–21^55^), with the optimizer ‘bobyqa’, and the function ‘glmmTMB’ of the package glmmTMB (version 1.0.2.1, 56). We included all identifiable random slopes^57,58^, which were manually dummy-coded and centered. To keep type I error rate at 5%, we compared all full models with a respective null model^59^ lacking only the test predictor but comprising the control predictors and complete random effects structure using a likelihood ratio test^60^. In case a higher order interaction term did not reveal significance, reduced models lacking that interaction term, but comprising all relevant lower order interactions, or main effect terms, were fitted. Model stability was assessed by comparing the estimates obtained from the model based on all data with those obtained from models with the levels of the random effects excluded one at a time. This revealed good model stability except for the model on SDB rates in pack-living dogs and wolves which indicates high uncertainty in results obtained from this model (Table S5) and warrants cautious interpretation. We performed parametric bootstrapping to obtain confidence intervals (function ‘bootMer’ of lme4) and assessed collinearity with the function ‘vif’ of the package car (version 3.0-0, 61), applied to a model lacking the random effects. This revealed no issues with collinearity in any of the models. For a detailed description of test and control predictors, as well as random effects included in fitted models, and all model output tables, see supplementary material.

A total of 104 samples 50 wolf and 54 pack dog samples were used for statistical analyses of urinary OTM concentrations across all test conditions. A total of 42 samples (20 wolf and 22 pack dog samples) were used for statistical analysis of OTM concentrations and 40 samples (19 wolf and 20 pack dog samples) were used for analysis of GCM concentrations following the social interaction tests. 204 human urine samples comprising 102 pairs of matched pre- and post-test samples were used for statistical analyses of human urinary OTM concentrations. A total of 67 pet dog urine samples were used for statistical analysis of urinary OTM concentrations and 61 for GCM concentrations.

### Ethics statement

This study was discussed and approved by the institutional Ethics and Animal Welfare Committee at the University of Veterinary Medicine Vienna, in accordance with Good Scientific Practice and ARRIVE guidelines and national as well as EU legislation (Protocol number ETK-05/03/2017). The human part was discussed and approved by the Ethics Committee at the Medical University of Vienna (Protocol number 1769/2017) and performed in accordance with the relevant guidelines and regulations. Written informed consent was obtained from each participant after full explanation of the nature of all procedures used. Written informed consent was also obtained from each participant to publish images and findings in an open-access publication.

## Results

### Experiment 1: Pack-living dogs and wolves

Effect of species and test condition on urinary oxytocin metabolite concentrations.

We found that urinary OTM concentrations were highest following the food condition, regardless of species (LMM; χ^2^ = 8.6, df = 3, P < 0.05; no interaction effect between species and condition: χ^2^ = 4.7, df = 3, P = 0.2; Table S3 and Figure S1). Male dogs had higher urinary OTM concentrations than female dogs and wolves (LMM; χ^2^ = 6.9, df = 1, P < 0.01; Table S3 and Figure S2), and feeding status (i.e. being fed or not the day before testing), relevant only for wolves (since dogs were fed daily) affected urinary OTM concentrations: being hungry resulted in higher OTM concentrations than being fed, regardless of test condition (LMM; χ^2^ = 11.6, df = 1, P < 0.01; Table S3).

Effect of species and relationship strength on body contact seeking and self-directed behaviors.

All dogs and all wolves approached and physically interacted with the bonded human partner, and all dogs also interacted with the familiar partner, but only 7 of 10 wolves did so. Overall, both pack dogs and wolves spent significantly more time in body contact with the bonded than the familiar partner (GLMM; χ^2^ = 12.8, df = 1, P < 0.01; no interaction effect of species and relationship strength; χ^2^ = 1.7, df = 1, P = 0.2) and there was no significant species difference in the duration the animals spent in body contact with the partners (GLMM; χ^2^ = 3.2, df = 1, P = 0.08) (Figure S3, Table S4; dogs: 87.7% of the interaction time with the bonded partner and 68.6% with the familiar partner; wolves: 72.9% with the bonded partner and 31.7% with the familiar partner). However, the coefficient of variation (CV) of wolves’ body contact seeking behavior was significantly higher than the dogs’ with the familiar partner (test statistic = 6.62, p-value ≤ 0.01; package cvequality, version 0.1. 3; 62). Hence, dogs’ body contact seeking behavior with the familiar partner was less variable than wolves’. Dogs displayed on average 1.4 SDBs (i.e., lip licks, yawns, body/head shakes) per minute of interaction time, whereas wolves only showed 0.5. In dogs, time spent in body contact with the familiar but not the bonded partner was positively associated with SDB rate (Figure S4 a), whereas in wolves a contrasting pattern emerged: time spent in body contact with the bonded partner was positively associated with SDB rate (GLMM; χ^2^ = 4.9, df = 1, P < 0.05) (Table S5, Figure S4 b). However, analysis of the model’s confidence intervals revealed considerable uncertainty associated with this result (Table S5).

Effect of species, relationship strength, and body contact on urinary oxytocin and glucocorticoid metabolite concentrations.

**Figure 2:**
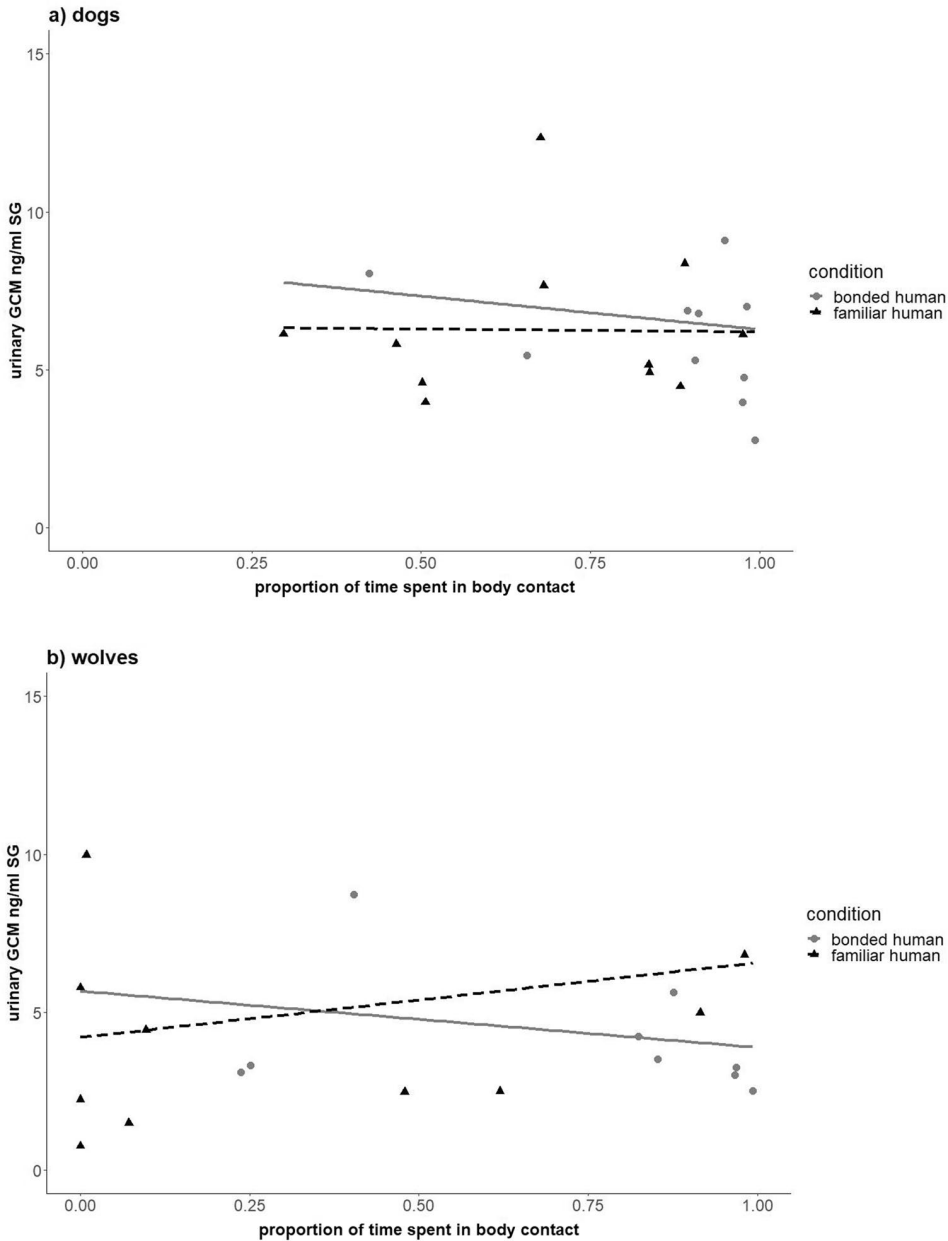
(**A**,**B**) Glucocorticoids and social contact with humans in pack dogs and wolves. Link between urinary glucocorticoid metabolites (GCM; ng/ml SG) concentrations, proportion of interaction time spent in body contact, and condition (i.e., relationship strength of the interaction partner: bonded = grey dots, solid line; familiar = black triangles; dotted line), in (**A**) pack-living dogs (N = 11; data points shown of all dogs in ‘familiar’ condition; data points shown of 10 dogs in ‘bonded’ condition due to insufficient urine sample volume for GCM measurement), and (**B**) wolves (N = 10; data points shown of all wolves in ‘familiar’ condition; data points shown of 9 wolves in ‘bonded’ condition due to insufficient urine sample volume for GCM measurement).

Next, we investigated whether dogs’ and wolves’ urinary OTM and GCM concentrations were differently affected by physical contact with a bonded or familiar partner. We found that both species had higher urinary OTM concentrations after interacting with the familiar than the bonded partner (LMM; χ^2^ = 5.5, df = 1, P < 0.05; Table S6), but there was no effect of physical contact on OTM concentrations in either species (LMM; χ^2^ = 0.84, df = 1, P = 0.36; Table S6). Dogs’ and wolves’ urinary GCM concentrations, however, correlated positively with time spent in physical contact with the familiar partner (LMM; χ^2^ = 6, df = 1, P < 0.05; Fig. 2 a-b; Table S7), and this seemed to be driven by the wolves (based on visual inspection of the data and the model estimates, Table S7) although the three-way interaction with species was not significant (LMM; χ^2^ = 2.4, df = 1, P = 0.12; Table S7). Finally, several control predictors had significant effects: individuals with higher baseline GCM concentrations also had higher GCM concentrations following the social interaction tests (LMM; χ^2^ = 16.4, df = 1, P < 0.001; Table S7), and being hungry resulted in higher GCM concentrations in wolves (LMM; χ^2^ = 7.9, df = 1, P < 0.001; Table S7). Overall, dogs had higher GCM concentrations than wolves (LMM; χ^2^ = 10, df = 1, P < 0.001; Table S7), regardless of condition.

Effect of species, relationship strength, and condition on human urinary oxytocin metabolite concentrations.

There was no effect of condition, time point (pre, post), relationship strength, or species (χ^2^ = 23.7, df = 19, P = 0.21) on human urinary OTM concentrations.

Taken together, results obtained from Experiment 1 were somewhat surprising: while we found increased OTM concentrations in dogs and wolves in response to food, in contrast to our predictions, no rise in OTM concentrations was evident following social contact with humans in either species (Figure S1). Instead, GCM concentrations correlated positively with time spent being petted by the familiar partner. Both species preferentially sought close contact with the bonded persons but wolves’ behavior was more varied than dogs’, partly in line with our predictions. SDBs were much more frequent in dogs, correlated positively with body contact, and were mediated by relationship strength. Finally, we found no effect of physical contact with a dog or a wolf on human urinary OTM concentrations.

### Experiment 2: Pet dogs

Effect of relationship strength on body contact seeking and self-directed behaviors.

All 10 pet dogs approached both human partners and spent on average 76.5% of the interaction time being petted by their owners, and 61.6% by the familiar person. Although they spent a smaller percentage of time in body contact with any human when there was a fence (64.8% with fence compared to 74.1% without fence), the difference was not statistically significant, and pet dogs did not differentiate between human partners based on relationship strength (GLMM; Full-Null model comparison: χ^2^ = 5.7, df = 2, P = 0.06; Figure S5). Like pack-living dogs, pet dogs displayed 1.4 SDBs per minute of interaction time, however, in contrast to the pack-living dogs, SDB rates were not affected by relationship strength, time spent in body contact, or fence presence (GLMM; Full-Null model comparison: χ^2^ = 7.0, df = 4, P = 0.13).

Effect of relationship strength and body contact on urinary oxytocin and glucocorticoid metabolite concentrations.

**Figure 3:**
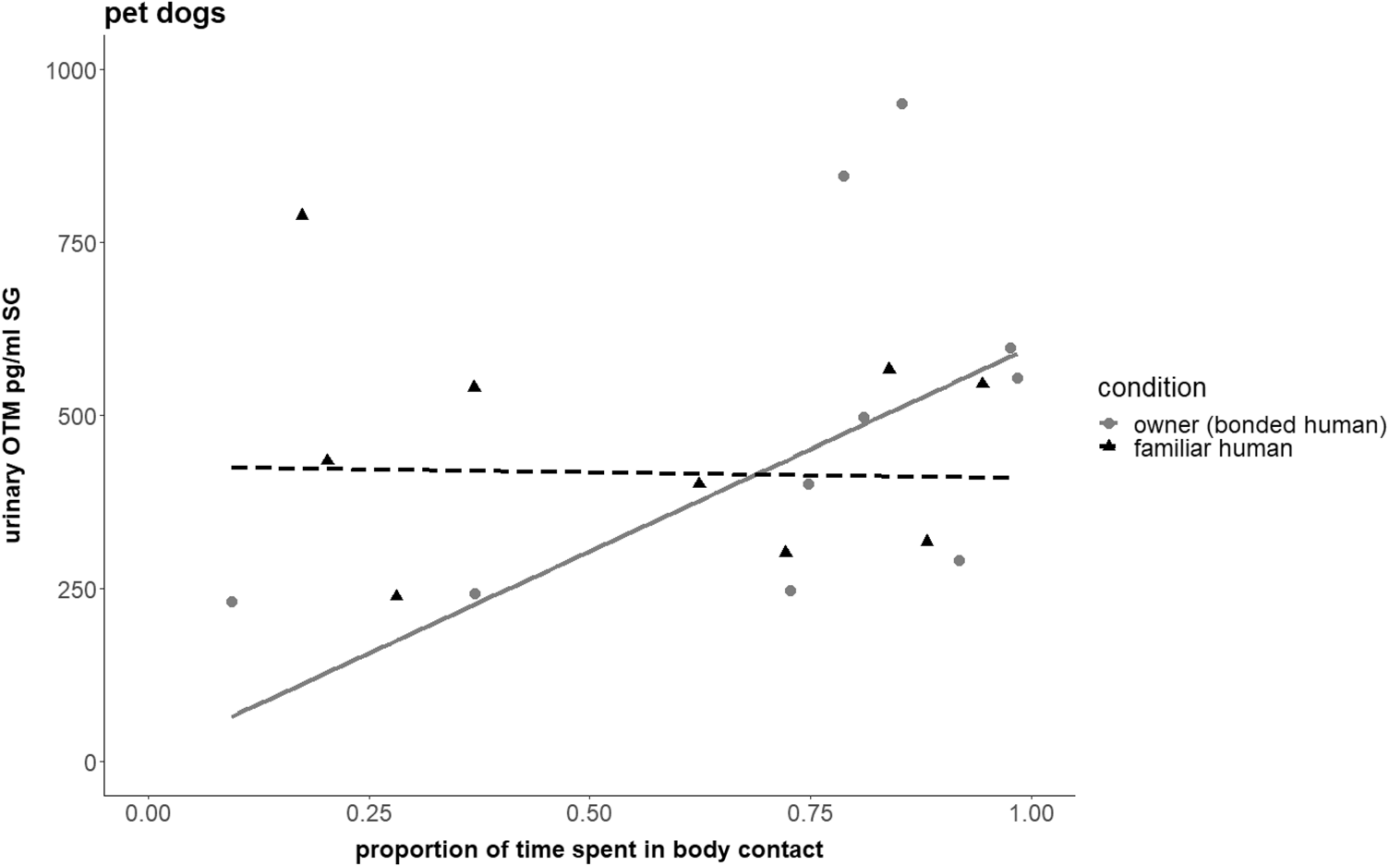
Oxytocin and social contact with humans in pet dogs. Link between urinary oxytocin metabolite (OTM; pg/ml SG) concentrations, proportion of interaction time spent in body contact, and condition (i.e., relationship strength of the interaction partner: owner/bonded = grey dots, solid line; familiar = black triangles; dotted line) in pet dogs (N = 10; data points shown of 9 pet dogs in ‘familiar’ condition due to unavailability of one dog for further tests; data points shown of all pet dogs in ‘bonded/owner’ condition). Figure 3 shows data points in ‘fence present’ condition for better comparison of pet dogs with pack-living dogs and wolves (see Figure S6 for data in ‘no fence’ condition).

In pet dogs, time spent in physical contact with their owners, but not the familiar person, was associated with higher urinary OTM concentrations in pet dogs (LMM; χ^2^ = 5.1, df = 1, P < 0.05; Table S9, Fig. 3). Further, pet dogs with higher basal OTM concentrations also had higher OTM concentrations following the interaction tests (LMM; χ^2^ = 5.5, df = 1, P < 0.05; Table S9), and the presence of the fence was associated with higher OTM concentrations (LMM; χ^2^ = 4.7, df = 1, P < 0.05; Table S9). No effect of body contact or relationship strength was evident for urinary GCM concentrations in pet dogs (LMM; Full-Null model comparison: χ^2^ = 6.1, df = 3, P = 0.11).

In line with our predictions we found that pet dogs’ urinary OTM concentrations correlated positively with the time spent in body contact with their owner but not the familiar partner (Fig. 3). No change in pet dogs’ urinary GCM concentrations was evident in either condition. Interestingly, while pet dogs interacted slightly longer with their owners than the familiar partners (Figure S5) this difference was not significant, and they showed the same rate of SDBs as the pack-living dogs but it was not affected by relationship strength or time spent in body contact.

## Discussion

Over the course of domestication, dogs may have developed an increased willingness to approach and interact closely with human partners compared to wolves (Hypersociability hypothesis:^3,17^), with an associated heightened oxytocin but decreased glucocorticoid release (‘Domestication Syndrome’:^4,6–9^). However, when comparing equally socialized pack dogs and wolves at the WSC, we did not find a strong difference in their human-directed contact seeking behavior: both species preferred the bonded partner over the familiar one, yet dogs spent overall more time with any human than wolves. Interestingly though, the behavior of the wolves was much more varied than the dogs’ when interacting with the familiar partner. Moreover, in contrast to the hypothesis that oxytocin release would drive affiliative interactions as well as be responsible for such behavioral differences between dogs and wolves, pack dogs’ and wolves’ OTM concentrations were unaffected by the petting received from either human partner. Both wolves and dogs had higher OTM concentrations following the food-only condition than after interacting with their hand raisers (Figure S1), suggesting that food is perceived as more rewarding than physical contact with humans. Taken together, the present study questions the idea that dogs’ increased human-directed sociability is due to domestication-induced alterations to the oxytocinergic system reactivity in response to human social contact.

Nevertheless, we found that pet dogs’ OTM concentrations correlated positively with being petted by their owner but not a familiar person (Fig. 3). Hence it seems that the nature of the dog-owner relationship differs from the relationship pack-living animals may establish with their hand-raisers and that previous experience with humans affects oxytocin release in dogs, lending support to the Two Stage hypothesis of domestication^34,35^. Accordingly, an individual’s ontogeny and life history plays an important role in the manifestation of behavioral inclinations (and physiological correlates) later in life (Table 1). The current findings suggest that this may also apply to experiencing close contact with humans as a rewarding event. This is further supported by pet dogs not responding with changes in their GCM concentrations to the interaction tests, while longer durations of physical contact with the familiar person led to higher GCM concentrations in the pack living animals, especially the wolves. Importantly, it was not the presence of the person, but rather the actual physical interaction which was associated with heightened GCM concentrations, since the shyest animals (that did not even approach the person) had lower concentrations than the ones that physically interacted with the familiar person. Increased glucocorticoid concentrations are associated with a perceived loss of control and predictability^63,64^, and, in dogs, have been shown to increase in response to an interaction with a stranger in a novel environment^65^. This indicates that physical contact with a non-bonded human activates the HPA axis in enclosure-living dogs and wolves, which may be interpreted as a sign that the animals perceived the situation as unpredictable and stressful (but, interestingly, still chose to stay close to the humans), whereas pet dogs did not react this way (probably due to more experience encountering, and being touched by, less familiar people). While dampened HPA axis reactivity has been proposed as a result of domestication mediating the behavioral fear response of domesticates (Emotional Reactivity and Selection for Tameness hypotheses^1,2^; ‘Domestication Syndrome’:^4^), our study rather indicates that this depends on the individuals’ socialization history and previous experience with different human interaction partners, at least with regard to circulating glucocorticoid concentrations.

Interestingly, and in stark contrast to predictions arising from the Emotional Reactivity hypothesis, SDB rates during the interaction tests were three times higher in dogs than wolves and positively related to time spent in body contact (mediated by bond strength) but not associated with GCM concentrations. Previously, SDBs have been interpreted mainly as stress signals^40,41^, but lip licking, in particular, is an important component of canid greeting behavior performed by the submissive towards the more dominant individual^66^. In human–dog interactions, lip licking may serve as communicative cue during greeting^24,67,68^ and may be used to avoid conflict in social situations perceived as mildly threatening^69^. Given dogs spent overall longer in physical contact with the humans, and pack and pet dogs performed SDBs at equally high rates it appears that rather than SDBs being an indicator of fear/stress, dogs behaved more submissively than wolves when closely interacting with humans. This finding lends tentative support to the notion that dogs were selected for increased submissive inclinations, ultimately resulting in more compliant cooperation partners for humans (Deferential Behavior hypothesis;^70^) but further studies are needed to test the significance of SDBs during human–dog interactions.

Further, we found that male pack-living dogs had significantly higher urinary OTM concentrations than female dogs and both male and female wolves (Figure S2; for previous discussion see^51^), but this sex difference was not significant in the pet dogs, although males (554.1 pg/ml SG) had on average higher OTM concentrations than females (506.1 pg/ml SG) and neutered individuals (333.6 pg/ml SG). Whereas all pack-living animals were hormonally intact (males were vasectomized at the age of 6 months to prevent reproduction but maintain the animals’ endocrine and behavioral profiles), half of the pet dogs in our sample were neutered. Both the HPA axis and the oxytocin system interact with and are modulated by gonadal hormone concentrations^71,72^. It is thus possible, that neutering could have affected pet dogs’ endocrine responses in the present study which should be investigated further in a larger sample.

We did not find an effect of interacting with a pack-living dog or wolf on urinary OTM concentrations in the human participants. While in contrast to our predictions, this finding is not entirely surprising. In fact, several studies report no effects of human–dog interactions on human OTM concentrations^27–29^. It is possible that peripheral samples (plasma, urine, saliva) and methods commonly used for measurement (enzyme immunoassay; EIA) are not sensitive enough to detect subtle effects, particularly if concentrations fall within the lower range of the assay^52^, as they do in human urine samples. Further, we could not control for cycle phase or hormonal contraceptives which are known to affect oxytocin concentrations in women^73,74^. In addition, we could not control what human participants did before donating the pre-test sample (although all were instructed not to eat or exercise before sampling). Thus, this could have confounded pre-test measures and prevented us from detecting a subsequent (subtle) change in human urinary OTM concentrations. Lastly, our study included only female human participants limiting the generalizability of the finding.

To conclude, given similar socialization and life experiences with humans, the comparison of pack dogs and wolves demonstrates that differences in social contact seeking behavior are subtle and mediated by relationship strength. No wolf/dog difference in urinary OTM/GCM concentrations associated with human contact was evident but in a follow-up experiment with pet dogs, physical contact with their owners was associated with higher urinary OTM concentrations. Results thus only partly support the notion of hypersocial dogs^3,17^. Rather, they are in line with the Two Stage hypothesis of domestication^34,35^, whereby not only phylogeny but also socialization and repeated interactions with humans predict wolves’ and dogs’ sociability and physiological correlates later in life. Accordingly, behavioral and hormonal species differences in response to human contact found in the present and previous studies likely reflect differences due to life experience rather than domestication. Further, by showing that life experience affects the manifestation of dogs’ social behavior and underlying hormonal correlates, our results are in line with epigenetic regulation of the endocrine system during the socialization process^75,76^.

## Supplementary Information

Supplementary Information 1.

Supplementary Information 2.

Supplementary Information 3.

Supplementary Video 1.

Supplementary Video 2.

Supplementary Video 3.

Supplementary Information 4.

## Data Availability

All data generated or analyzed during this study are included in the Supplementary Information files.
