# Supplementary material for: Life experience rather than domestication accounts for dogs’ increased oxytocin release during social contact with humans
Source: Sci Rep. 2021 Jul 13;11:14423. doi: 10.1038/s41598-021-93922-1 (PMC8277847; doi:10.1038/s41598-021-93922-1)
Supplement: Supplementary file 7 — Supplementary Information 4. [file 41598_2021_93922_MOESM7_ESM.pdf]

Supplementary Materials for

Life experience rather than domestication accounts for dogs' increased oxytocin release during social contact with humans

Gwendolyn Wirobski *, Friederike Range, Franka S. Schaebs, Rupert Palme, Tobias Deschner, Sarah Marshall-Pescini

*Corresponding author. Email: gwendolyn.wirobski@vetmeduni.ac.at, gwen.wirobski@gmail.com

This PDF file includes:

Supplementary Text
Figs. S1 to S6
Tables S1 to S9

Other Supplementary Materials for this manuscript include the following:
Movies S1 to S3
Datasets S1 to S3

Supplementary Text

Detailed description of all statistical models

Experiment 1: Pack-living dogs and wolves

1. *Effect of species and condition on urinary oxytocin metabolite concentrations*

To examine if domestication has altered dogs' urinary OTM concentrations in response to social interactions with humans, we tested whether test condition affected urinary OTM concentrations differently in pack-living dogs and wolves. To this end, we fitted a Linear Mixed Model (LMM) with Gaussian error structure. The response variable (urinary OTM pg/ml SG) was log transformed to obtain normally distributed and homogenous residuals. The test predictor was the interaction between condition (factor with four levels: 'bonded human', 'familiar human', 'food control', 'baseline') and species (factor with two levels: 'wolf', 'dog'), and their respective main effects. To account for species-specific sex differences we included the sex by species interaction. Feeding status (factor with two levels, 'fed' and 'not fed' the day before testing) and reproductive phase (factor with two levels: 'anestrus', 'diestrus'; testing during proestrus and estrus was avoided) were inserted as control variables (reproductive phase was subsequently dropped from further OTM models because it did not affect OTM concentrations). Random effects of subject, pack, and assay plate were included to account for repeated sampling and variation across packs and plates.

2. *Effect of species and relationship strength on body contact seeking and self-directed behaviors*

First, to investigate whether relationship strength positively affected body contact seeking in dogs and wolves, we fitted a beta model using the R package glmmTMB to a subset of the data comprising the social interaction conditions. The response variable was the normalized duration (proportion of interaction time the animal was visible on camera) of interaction time spent in body contact with the human partner. The test predictor was the interaction between relationship strength (i.e., factor with two levels: 'bonded' and 'familiar') and species. Sex was included as a control predictor and subject, pack, and interaction partner identity were included as random effects. To compare the species-specific variability in contact seeking behavior of dogs and wolves, we calculated the coefficients of variation (CV) using the R package cvequality.

To test whether self-directed behaviors (sum of yawning, lip licking, head/body shakes) differed between dogs and wolves, according to relationship strength of the interaction partner, and whether they were associated with body contact, we fitted a generalized linear mixed model (GLMM) with Poisson error distribution. The test predictor was the three-way interaction between relationship strength (i.e., factor with two levels: 'bonded' and 'familiar'), species, and normalized duration spent in body contact. Sex was included as a control predictor and subject, pack, and interaction partner identity were included as random effects. Time in sight was included as an offset term to account for small differences in the durations that the animals were visible on camera.

3. *Effect of species, relationship strength, and body contact on urinary oxytocin metabolite concentrations*

We fitted another model to specifically investigate the effects of species, relationship strength, and body contact on urinary OTM concentrations following the interaction tests. The response variable was urinary OTM (pg/ml SG) which was log transformed (see above), and the test predictor was the three-way-interaction of species, proportion time spent in body contact, and relationship strength (i.e., factor with two levels: 'bonded' and 'familiar'). Control predictors were feeding status, the interaction between species and sex, and the frequency of self-directed behaviors (SDBs; sum of lips licking, yawning, body shaking divided by seconds in sight) performed by the animals during the interaction tests and their subsequent urinary GCM concentrations (co-variate, z-transformed) as a proxy for stress/arousal. Basal OTM concentrations (i.e., OTM concentrations on resting days where no tests took place; 'baseline' samples) were included as a co-variate to control for individual variation in OT system activity (z-transformed). Random effects of subject, pack, partner identity, and assay plate were included.

4. *Effect of species, relationship strength, and body contact on urinary glucocorticoid metabolite concentrations*

To test the link between body contact, relationship strength, and urinary GCM concentrations in dogs and wolves, we fitted one more LMM. The response variable was urinary GCM (ng/ml SG) which was log transformed (see above), and the test predictor was the three-way-interaction of species, proportion time spent in body contact, and relationship strength (factor with two levels, 'bonded' and 'familiar'). We controlled for feeding status, reproductive phase, and the interaction between species and sex, as these factors have been shown to affect GCM concentrations in a previous study in this population (63). The frequency of SDBs (i.e., sum of lips licking, yawning, body shaking divided by seconds in sight) performed by the animals during the interaction tests was included as a co-variate. Baseline GCM concentrations were included to account for individual differences (z-transformed). Random effects of subject, pack, and partner identity were included.

<u>Humans</u>

1. *Effect of species, relationship strength, and condition on human urinary oxytocin metabolite concentrations*

To investigate urinary OTM concentrations in the human participants, we fitted a LMM with log transformed OTM concentrations as the response variable, and a four-way interaction between condition (factor with two levels: 'social interaction' and 'control'), species ('dog', 'wolf'), relationship strength ('bonded (hand raiser)', 'familiar'), and time of sample ('pre', 'post'-testing). Random effects of subject, animal partner, dyad, and plate were included.

Experiment 2: Pet dogs

1. *Effect of relationship strength on body contact seeking and self-directed behaviors*

To first examine if relationship strength (i.e., factor with two levels: ’owner’ and ‘familiar’) affected time spent in body contact in pet dogs, we fitted a beta model (package glmTMBB) analogous to the one in the pack-living animals. We controlled for sex (factor with three levels: ‘male’, ‘female’, ‘castrated/spayed’) and the presence of a fence (factor with two levels, ‘fence present’ and ‘no fence present’) between the human interaction partner and the dog. Random effects of subject and partner identity were included. To investigate the effect of relationship strength and body contact on the sum of SDBs in pet dogs, we fitted a GLMM with Poisson error distribution, analogous to the one in the pack animals (see above). We included the interaction between relationship strength of the partner and time spent in body contact as test predictor, sex and fence as control predictors, and random effects of subject and partner identity. Time in sight was included as an offset term to account for differences in visibility on camera.

2. *Effect of relationship strength and body contact on urinary oxytocin and glucocorticoid metabolite concentrations*

To investigate whether urinary OTM and GCM concentrations were affected by relationship strength and the time spent in body contact, we fitted two LMMs with Gaussian error structures (using log-transformed OTM and GCM concentrations as response variables). We included sex, fence, and the frequency of SDBs (z-transformed co-variate) as control factors. Basal OTM and GCM concentrations (i.e., OTM / GCM concentrations on resting days where no testing took place) were included as co-variates to control for individual variation in unstimulated OT/GC system activity. Random effects of subject and partner identity, and plate were included.

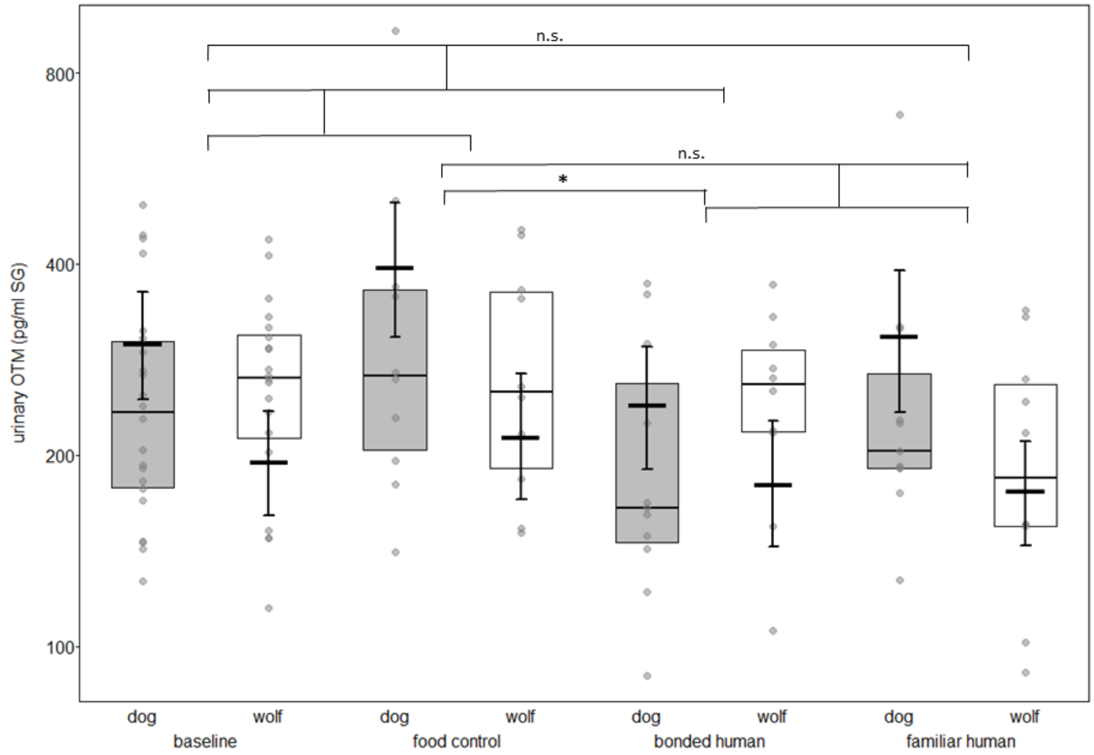

Fig.S1: Urinary OTM concentrations (pg/ml SG) across all four conditions in pack-living dogs (N=11; grey boxes) and wolves (N=10; white boxes). Indicated are medians and quartiles (thin horizontal lines with boxes) as well as the fitted model and its 95% confidence intervals (thick horizontal lines with error bars). * $P \leq 0.05$.

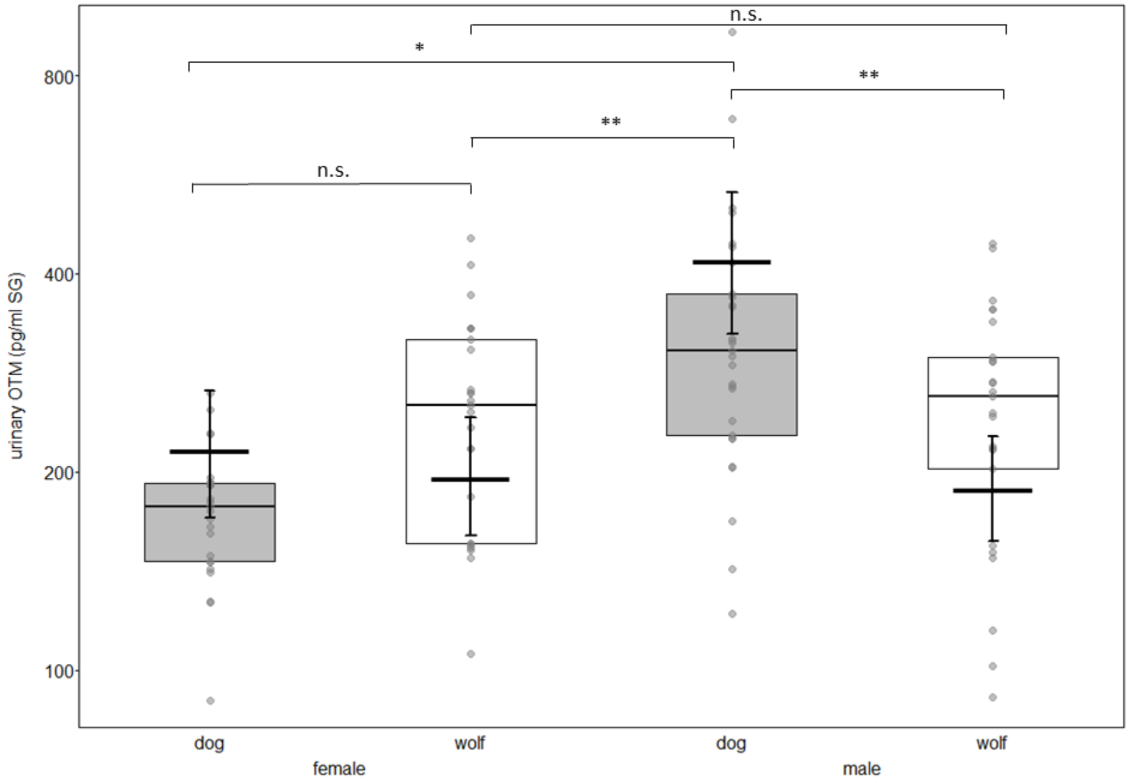

Fig. S2: Effect of sex on urinary OTM concentrations in dogs (N=11; grey boxes) and wolves (N=10; white boxes). Indicated are medians and quartiles (thin horizontal lines with boxes) as well as the fitted model and its 95% confidence intervals (thick horizontal lines with error bars). ** $P \leq 0.01$, * $P \leq 0.05$.

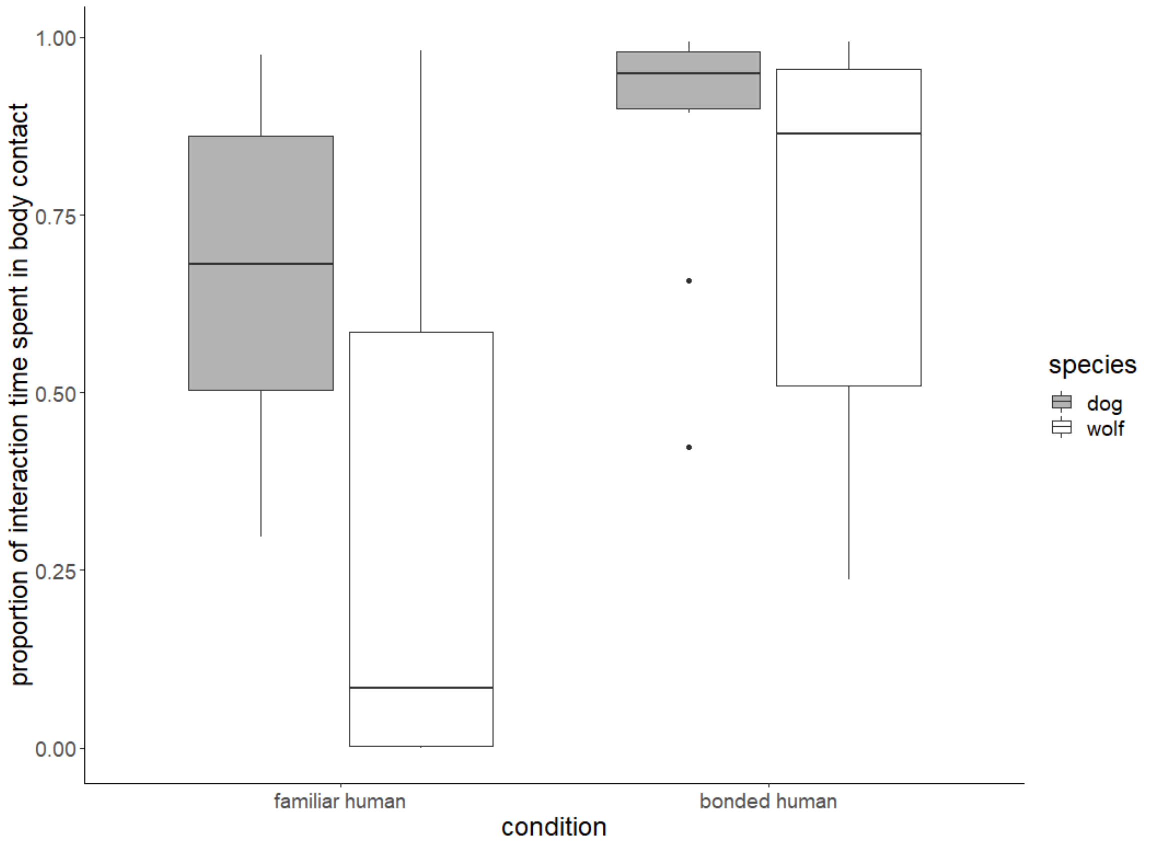

Fig. S3: Proportion of interaction time spent in body contact with the human partner, mediated by condition (i.e., relationship strength: ‘familiar’, ‘bonded’) and species (grey = dog, white = wolf). Indicated are medians and quartiles (thin horizontal lines and boxes).

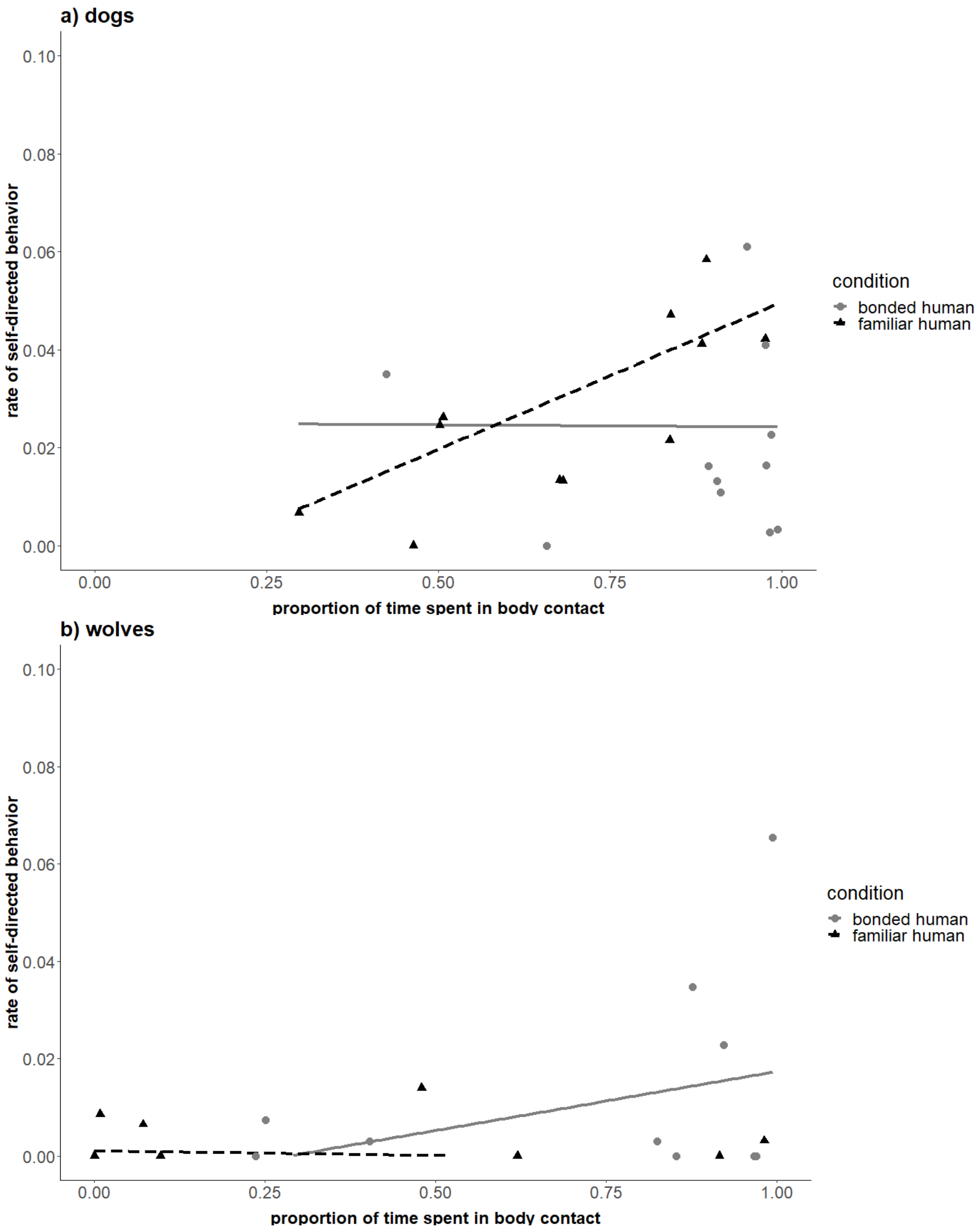

Fig. S4 a - b: Association between rate of self-directed behavior per second of interaction time and proportion of interaction time spent in body contact, by species (a = dogs; b = wolves) and relationship strength of the interaction partner (bonded = grey dots, solid line; familiar = black triangles; dotted line).

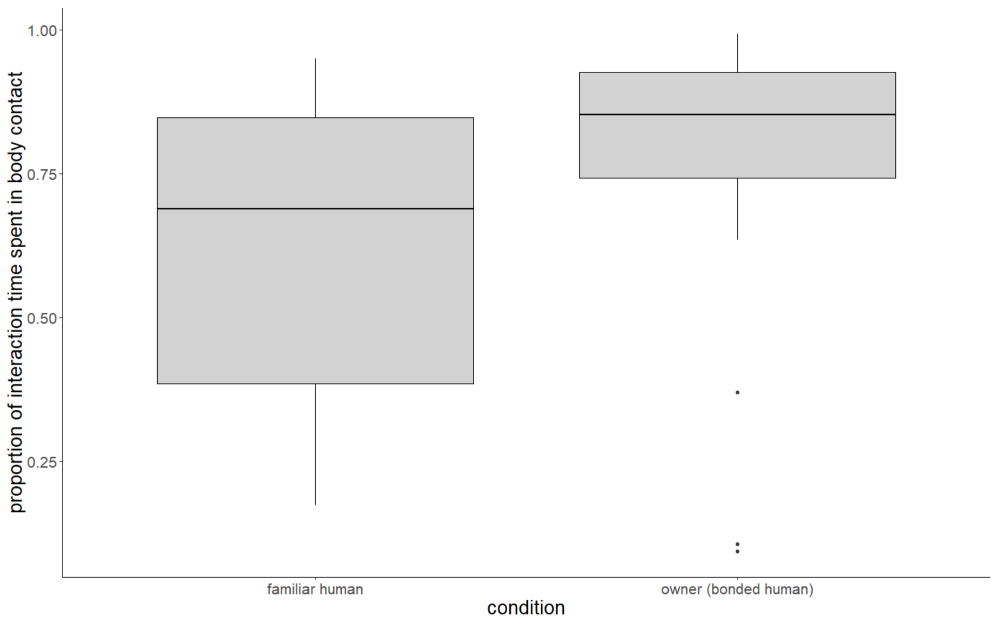

Fig. S5: Proportion of interaction time spent in body contact with the human partners, mediated by condition (i.e., relationship strength, familiar and bonded/owner) in pet dogs. Indicated are medians and quartiles (thin horizontal lines and boxes).

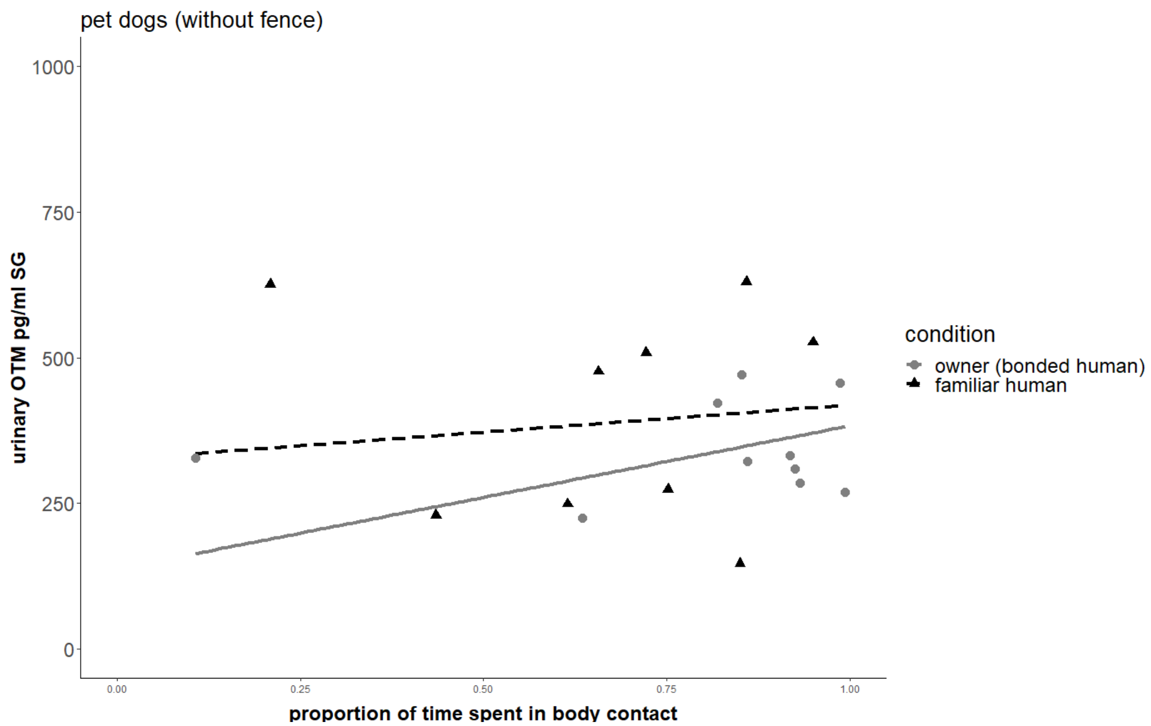

Fig. S6: Association between urinary OTM (pg/ml SG) concentrations, proportion of interaction time spent in body contact, and condition (i.e., relationship strength of the interaction partner: owner/bonded = grey dots, solid line; familiar = black triangles; dotted line) in **pet dogs (**N=10; data points shown of 9 pet dogs in 'familiar' condition due to unavailability of one dog for further tests; data points shown of all pet dogs in 'owner/bonded' condition) without a fence present between the dogs and the human interaction partners.

Table S1: Details of the pack-living dogs and wolves used in Experiment 1.

**Animal ID**	**Species**	**Sex**	**Age** (at study begin; in years)
Amarok	Wolf	Male	5
Aragorn	Wolf	Male	9
Tekoa	Wolf	Male	2
Geronimo	Wolf	Male	8
Nanuk	Wolf	Male	9
Tala	Wolf	Female	5
Yukon	Wolf	Female	8
Shima	Wolf	Female	9
Taima	Wolf	Female	2
Una	Wolf	Female	5
Meru	Dog	Male	7
Maisha	Dog	Male	8
Hiari	Dog	Male	3
Enzi	Dog	Male	3
Asali	Dog	Male	8
Sahibu	Dog	Male	3
Bora	Dog	Female	7
Binti	Dog	Female	7
Panya	Dog	Female	3
Imara	Dog	Female	3
Nia	Dog	Female	6

Table S2: Relevant subset of the ethogram used for video coding and subsequent statistical analyses of behavioral correlates during the human-animal interaction tests.

OOS (duration)	Out of sight; animal not visible on the video
Proximity to human (duration)	During interaction or training: subject is staying within one body length of the human partner
Yawning (event)	Wide opening of the jaws, without vocalizing
Body / head shaking (event)	Shaking the body or head from side to side
Lip licking (event)	Briefly extruding the tongue from the mouth and running it over the animal's own lips/nose
Body contact (duration)	Touching the animal in a gentle way, on the side of its body or head

Table S3: Full and reduced model outputs for urinary OTM (pg/ml SG; log transformed) concentrations across all test conditions.

**Full model** **Predictor**	**Estimate**	**SE**	**Df**	**χ 2**	**P**
Intercept	5.530	0.155	NA*	NA*	NA*
Species[†]	0.079	0.191	NA*	NA*	NA*
Food control condition[‡]	0.499	0.106	NA*	NA*	NA*
Familiar human condition[‡]	0.252	0.136	NA*	NA*	NA*
Baseline condition[‡]	0.220	0.094	NA*	NA*	NA*
Sex[§]	0.662	0.139	1	NA*	NA*
Feeding status[¶]	-0.597	0.096	1	12.513	**0.000**
Reproductive phase[#]	-0.014	0.078	1	0.017	0.895
Species[†]: Food control condition[‡]	-0.327	0.143	3	4.650	0.199
Species[†] : Familiar human condition[‡]	-0.276	0.186	NA*	NA*	NA*
Species[†] : Baseline condition[‡]	-0.139	0.133	NA*	NA*	NA*
Species[†] : Sex[§]	-0.703	0.185	1	9.029	**0.003**

**Reduced model** **Predictor**	**Estimate**	**SE**	**Df**	**χ 2**	**P**	**Lower CI**	**Upper CI**
(Intercept)	5.651	0.148	NA*	NA*	NA*	5.314	5.981
Species[†] : Sex[§]	-0.724	0.188	1	6.926	**0.008**	-1.166	-0.255
Food control condition[‡]	0.332	0.085	3	8.605	**0.035**	0.151	0.514
Familiar human condition[‡]	0.131	0.114	NA*	NA*	NA*	-0.121	0.369
Baseline condition[‡]	0.157	0.073	NA*	NA*	NA*	-0.005	0.322
Reproductive phase[#]	-0.033	0.080	1	0.092	0.762	-0.246	0.166
Feeding status[¶]	-0.619	0.104	1	11.588	**0.001**	-0.850	-0.407

Statistically significant results ($P \leq 0.05$) appear in bold. SE, standard error. Df, degrees of freedom. CI, confidence interval. [*] Not shown because of limited interpretation only.

[†, ‡, §, ¶, #] χ^2 and P values refer to comparison with the test predictors' reference levels:

[†] Reference level 'dog'.

[‡] Reference level 'bonded human condition'.

[§] Reference level 'female'.

[¶] Reference level 'not fed/fasted'.

[#] Reference level 'anestrus'.

Table S4: Full and reduced model outputs for time spent in body contact (as proportion of interaction time) during social interaction tests.

**Full model**	**Estimate**	**SE**	**Df**	**χ 2**	**P**		
**Predictor**							
Intercept	2.154	0.701	NA*	NA*	NA*		
Species[†]	-0.859	0.912	NA*	NA*	NA*		
Relationship strength[‡]	-1.442	0.623	NA*	NA*	NA*		
Sex[§]	0.114	0.450	1	0.066	0.798		
Species[†] : Relationship strength[‡]	-1.180	0.859	1	1.663	0.197		
**Reduced model**	**Estimate**	**SE**	**Df**	**χ 2**	**P**	**Lower CI**	**Upper CI**
**Predictor**							
(Intercept)	2.506	0.659	NA*	NA*	NA*	1.283	3.766
Species[†]	-1.483	0.784	1	3.169	0.075	-2.918	-0.054
Relationship strength[‡]	-2.066	0.483	1	12.818	**0.000**	-3.019	-1.157
Sex[§]	0.105	0.458	1	0.054	0.817	-0.803	1.006

Statistically significant results ($P \leq 0.05$) appear in bold. SE, standard error. Df, degrees of freedom. CI, confidence interval. [*] Not shown because of limited interpretation only.
[†, ‡, §] χ^2 and P values refer to comparison with the test predictors' reference levels:
[†] Reference level 'dog'.
[‡] Reference level 'bonded'.
[§] Reference level 'female'.

Table S5: Full model output for rate of self-directed behaviors during social interaction tests.

Full model Predictor	Estimate	SE	Df	χ^2	P	Lower CI	Upper CI
Intercept	-3.293	1.490	NA*	NA*	NA*	-6.980	-0.588
Species[†]	-3.616	2.013	NA*	NA*	NA*	-10.411	0.516
Relationship strength[‡]	-2.250	1.517	NA*	NA*	NA*	-5.106	0.808
Body contact[¶]	-1.007	1.687	NA*	NA*	NA*	-4.175	2.656
Sex[§]	0.191	0.468	1	0.173	0.677	-0.593	1.069
Species[†] : Relationship strength[‡]	3.630	2.073	NA*	NA*	NA*	-0.869	10.398
Species[†] : Body contact[¶]	3.270	2.362	NA*	NA*	NA*	-1.355	10.769
Relationship strength[‡] : Body contact[¶]	3.338	1.782	NA*	NA*	NA*	-0.035	6.835
Species[†] : Relationship strength[‡] : Body contact[¶]	-6.496	2.701	1	4.935	**0.026**	-17.721	-1.737

Statistically significant results ($P \leq 0.05$) appear in bold. SE, standard error. Df, degrees of freedom. CI, confidence interval. [*] Not shown because of limited interpretation only.
[†, ‡, §] χ^2 and P values refer to comparison with the test predictors' reference levels:
[†] Reference level 'dog'.
[‡] Reference level 'bonded'.
[§] Reference level 'female'.
[¶] Normalized duration (z-transformed).

Table S6: Full and reduced model outputs for urinary OTM (pg/ml SG; log transformed) concentrations for social interaction tests.

**Full model**<br>**Predictor**	**Estimate**	**SE**	**Df**	**χ^2**	**P**
Intercept	4.270	0.537	NA*	NA*	NA*
Species[†]	0.182	0.613	NA*	NA*	NA*
Sex[§]	0.508	0.202	NA*	NA*	NA*
SDB rate[‖]	-8.170	3.574	1	4.007	**0.045**
Body contact[#]	-0.097	0.549	NA*	NA*	NA*
Relationship strength[‡]	0.362	0.566	NA*	NA*	NA*
Feeding status[¶]	0.305	0.133	1	4.196	**0.041**
Basal OTM concentration[**]	0.036	0.070	1	0.251	0.616
GCM concentration[††]	0.030	0.063	1	0.216	0.642
Species[†] : Sex[§]	-0.725	0.267	1	6.705	**0.010**
Species[†] : Body contact[#]	0.465	0.635	1	0.192	0.661
Species[†] : Relationship strength[‡]	-0.013	0.632	1	1.988	0.159
Body contact[#] : Relationship strength[‡]	0.219	0.696	1	0.186	0.667
Species[†] : Relationship strength[‡] : Body contact[#]	-0.501	0.837	1	0.330	0.565

**Reduced model**<br>**Predictor**	**Estimate**	**SE**	**Df**	**χ^2**	**P**	**Lower CI**	**Upper CI**
Intercept	4.031	0.299	NA*	NA*	NA*	3.263	4.847
Body contact[#]	0.224	0.199	1	0.836	0.361	-0.332	0.816
Relationship strength[‡]	0.417	0.118	1	5.463	**0.019**	0.130	0.721
Basal OTM concentration[**]	0.037	0.070	1	0.244	0.621	-0.152	0.209
GCM concentration[††]	0.017	0.063	1	0.066	0.798	-0.145	0.173
Species[†] : Sex[§]	-0.778	0.276	1	7.033	**0.008**	-1.413	-0.065
SDB rate[‖]	-6.202	3.259	1	2.980	0.084	-15.266	2.290
Feeding status[¶]	0.288	0.143	1	2.701	0.100	-0.060	0.619

Statistically significant results ($P \leq 0.05$) appear in bold. SE, standard error. Df, degrees of freedom. CI, confidence interval. [*] Not shown because of limited interpretation only.

[†, ‡, §, ¶] χ^2 and P values refer to comparison with the test predictors' reference levels:

[†] Reference level 'dog'.

[‡] Reference level 'bonded'.

[§] Reference level 'female'.

[¶] Reference level 'not fed/fasted'.

[#] Normalized duration (z-transformed).

[‖] Normalized for time visible on camera (z-transformed).

[**] Urinary OTM (pg/ml SG) concentration on non-testing days ('baseline'; z-transformed).

[††] Urinary GCM (ng/ml SG) concentration (following social interaction test; z-transformed).

Table S7: Full and reduced model outputs for urinary GCM (ng/ml SG; log transformed) concentrations for social interaction tests.

**Full model**<br>**Predictor**	**Estimate**	**SE**	**Df**	**χ 2**	**P**
Intercept	2.330	0.424	NA*	NA*	NA*
Species†	-0.509	0.477	NA*	NA*	NA*
Sex§	-0.060	0.156	NA*	NA*	NA*
SDB rate$^{\Vert}$	3.882	2.698	1	1.337	0.247
Body contact$^{\#}$	-0.312	0.443	NA*	NA*	NA*
Relationship strength‡	-0.232	0.412	NA*	NA*	NA*
Feeding status¶	-0.443	0.107	1	11.615	**0.001**
Reproductive phase††	0.089	0.112	1	0.532	0.466
Basal GCM concentration**	0.324	0.053	1	16.386	**0.000**
Species† : Sex§	0.154	0.232	1	0.421	0.517
Species† : Body contact$^{\#}$	-0.245	0.523	1	1.051	0.305
Species† : Relationship strength‡	-0.412	0.468	1	3.582	0.058
Species†: Relationship strength‡ : Body contact$^{\#}$	1.206	0.620	1	2.438	0.118

**Reduced model**<br>**Predictor**	**Estimate**	**SE**	**Df**	**χ 2**	**P**	**Lower CI**	**Upper CI**
Intercept	2.272	0.255	NA*	NA*	NA*	1.776	2.821
Body contact$^{\#}$: Relationship strength‡	0.797	0.278	1	5.969	**0.015**	0.175	1.376
Species†	-0.504	0.142	1	10.018	**0.002**	-0.819	-0.230
Basal GCM concentration**	0.307	0.051	1	16.432	**0.000**	0.193	0.422
Reproductive phase††	0.060	0.116	1	0.221	0.638	-0.186	0.324
Sex§	0.049	0.112	1	0.189	0.664	-0.156	0.262
SDB rate$^{\Vert}$	-0.415	2.590	1	0.025	0.875	-6.538	4.739
Feeding status¶	-0.407	0.128	1	7.855	**0.005**	-0.665	-0.150

Statistically significant results ($P \leq 0.05$) appear in bold. SE, standard error. Df, degrees of freedom. CI, confidence interval. * Not shown because of limited interpretation only.

$^{\dagger, \ddagger, \S, \P, \dagger\dagger}$ χ^2 and P values refer to comparison with the test predictors' reference levels:

† Reference level 'dog'.

‡ Reference level 'bonded'.

§ Reference level 'female'.

¶ Reference level 'not fed/fasted'.

†† Reference level 'anestrus'.

$^{\#}$ Normalized duration (z-transformed).

$^{\Vert}$ Normalized for time visible on camera (z-transformed).

** Urinary GCM (ng/ml SG) concentration on non-testing days ('baseline'; z-transformed).

Table S8: Details of the pet dogs used in Experiment 2.

**Dog ID**	**Breed**	**Sex**	**Age** (at study begin; in years)
Coco	Border Collie	Female	2
Miley	Border Collie	Female	9
Casey	Chihuahua	Female (spayed)	8
Leni	Mix	Female (spayed)	5
Olive	Mix	Female (spayed)	12
Orion	Mix	Male	1
Pepeo	Mix	Male	5
Mago	Golden Retriever	Male	14
Frodo	Mix	Male (castrated)	5
Hakima	Mix	Male (castrated)	8

Table S9: Full model output for urinary OTM (pg/ml SG; log transformed) concentrations for social interaction tests in pet dogs.

**Full model**<br>**Predictor**	**Estimate**	**SE**	**Df**	**χ 2**	**P**	**Lower CI**	**Upper CI**
Intercept	5.873	0.179	NA*	NA*	NA*	5.455	6.224
Body contact[#] : Relationship strength[‡]	0.797	0.337	1	5.138	**0.023**	-0.033	1.582
Basal OTM concentration[**]	0.217	0.085	1	5.530	**0.019**	0.029	0.396
Sex female[§]	-0.095	0.187	2	4.529	0.842	-0.506	0.350
Sex male[§]	0.218	0.157	2	4.529	0.629	-0.113	0.587
Fence[¶]	-0.283	0.119	1	4.716	**0.030**	-0.534	-0.025
SDB rate[‖]	1.179	2.709	1	0.172	0.678	-4.628	7.235

Statistically significant results ($P \leq 0.05$) appear in bold. SE, standard error. Df, degrees of freedom. CI, confidence interval. [*] Not shown because of limited interpretation only.
[‡, ¶] χ^2 and P values refer to comparison with the test predictors' reference levels:
[‡] Reference level 'familiar'.
[¶] Reference level 'fence present'.
[§] Reference level 'castrated/spayed';
P values for each level of the factor 'sex' were obtained from post hoc pairwise comparisons (R function lsmeans).
[#] Normalized duration (z-transformed).
[‖] Normalized for time visible on camera (z-transformed).
[**] Urinary OTM (pg/ml SG) concentration on non-testing days ('baseline'; z-transformed).

Movie S1. Dyadic social interaction test with a hand-raised, pack-living wolf.

Movie S2. Dyadic social interaction test with a hand-raised, pack-living dog.

Movie S3. Dyadic social interaction test with a pet dog.

Dataset S1. Data collected from pack-living dogs and wolves.

Dataset S2. Data collected from human participants.

Dataset S3. Data collected from pet dogs.
